# Sign language recognition based on dual-path background erasure convolutional neural network

**DOI:** 10.1038/s41598-024-62008-z

**Published:** 2024-05-18

**Authors:** Junming Zhang, Xiaolong Bu, Yushuai Wang, Hao Dong, Yu Zhang, Haitao Wu

**Affiliations:** 1 School of Computer and Artificial Intelligence, Huanghuai University, Zhumadian, 463000 Henan Province China; 2 Key Laboratory of Intelligent Lighting, Henan Province, Zhumadian, 463000 China; 3 School of Computer Science, Zhongyuan University of Technology, Xinzheng, 450007 Henan China

**Keywords:** Electrical and electronic engineering, Mechanical engineering

## Abstract

Sign language is an important way to provide expression information to people with hearing and speaking disabilities. Therefore, sign language recognition has always been a very important research topic. However, many sign language recognition systems currently require complex deep models and rely on expensive sensors, which limits the application scenarios of sign language recognition. To address this issue, based on computer vision, this study proposed a lightweight, dual-path background erasing deep convolutional neural network (DPCNN) model for sign language recognition. The DPCNN consists of two paths. One path is used to learn the overall features, while the other path learns the background features. The background features are gradually subtracted from the overall features to obtain an effective representation of hand features. Then, these features are flatten into a one-dimensional layer, and pass through a fully connected layer with an output unit of 128. Finally, use a fully connected layer with an output unit of 24 as the output layer. Based on the ASL Finger Spelling dataset, the total accuracy and Macro-F1 scores of the proposed method is 99.52% and 0.997, respectively. More importantly, the proposed method can be applied to small terminals, thereby improving the application scenarios of sign language recognition. Through experimental comparison, the dual path background erasure network model proposed in this paper has better generalization ability.

## Introduction

Verbal communication is an important part of interpersonal communication, which is not only a tool for transferring information, but also a bridge for mutual understanding, respect, and tolerance between people^1,2^. However, some special populations are unable to communicate through language or understand the verbal expressions of others^3^. These people may suffer from congenital or acquired disabilities that hinder their ability to communicate verbally, and sign language becomes a tool for them to communicate^4^. Sign languages include several types, and each one requires time to learn and practice to master. For most people, they may never be exposed to the sign language aspect in their lifetime, and it is even more difficult to master one type of sign language. Therefore, even though this particular group of people already has the tools to carry out communication, it is limited to the interior of this group^5^. With the development of Artificial Intelligence, gesture recognition technology provides a way for ordinary people to understand gestures as well^6^.

Until now, a variety of techniques exist that enable gesture recognition^7^, including vision-based gesture recognition, smart glove-based gesture recognition^8^, gesture recognition based on partial least squares regression^10^, gesture recognition based on FMCW radar^11^, and gesture recognition based on Kinect sensors^12^. Tompson et al.^13^ utilized a convolutional network to extract features and generate heat maps indicating joint points. They employed inverse kinematics methods to infer hand postures based on the extracted features and heat maps. Cao et al.^14^ proposed a gesture recognition method that integrates multiple image features and multi-core learning support vector machines (SVM). Gestures of unknown categories can be recognized by the trained multi-core SVM, and a higher recognition rate is obtained than the traditional single-core classifier. Sadeddine et al.^15^ used gradient local autocorrelation (GLAC), fast discrete curve transform (FDCT) and Gabor wavelet transform methods to extract gesture features. SqueezeNet proposed by Iandola et al.^16^ greatly reduces the number of parameters of the model while maintaining prediction accuracy. Howard et al.^17^ proposed a class of efficient lightweight deep neural network models called MobileNets based on a streamlined architecture for mobile and embedded vision applications. Sandler et al.^18^ proposed a new mobile architecture MobileNetV2, which is based on the direction residual structure and significantly reduces the memory footprint of the inference process. Zimmermann et al.^19^ employed the HandSegNet segmentation network to locate the hand area, followed by image cropping and scaling based on the hand mask. Chen et al.^20^ proposed the nonparametric structure regularization Machine (NSRM) for two-dimensional hand pose estimation. Kwolek et al.^21^ presented a gesture recognition method that utilized RGB images. They employed a generative adversarial network and ResNet model for gesture segmentation and recognition. Huang Jie et al.^22^ combined 3D convolution with an attention mechanism for sign language recognition. However, 3D convolution has the drawback of increased parameter count and longer training time compared to 2D convolution. Additionally, researchers^23–25^ combined convolutional neural networks with recurrent neural networks for sign language recognition tasks. However, the network structure combining convolutional and recurrent networks is complex, leading to long training times and reduced recognition efficiency. Pu et al.^26^ employed multimodal inputs to enhance sign language recognition rates. However, compared to RGB images, modal acquisition methods such as depth images, optical flow images, and skeleton images are more complex, and the image processing involved is relatively challenging. Liu et al.^27^ proposed a temporal decoupled graph convolution network (TD-GCN), which is a skeleton-based gesture recognition method, and TD-GCN effectively improves the modeling ability of GCN. Jin et al.^28^ proposed a dynamic gesture recognition method based on CNN-Transformer network to solve the problem of misrecognition in a random dynamic interference environment, and obtained relatively good results on the data set they constructed including six dynamic gestures and two random interferences. Xie et al.^29^ obtain multi-scale visual features by sampling video segments at different frame rates, and recognize sign language through feature fusion through attention mechanism. Yang et al.^30^ proposed a gesture recognition system using frequency shift keying (FSK) radar. The system uses a convolutional neural network model to recognize gestures within a certain range, breaking the limitations of gesture recognition at a fixed distance.

These technologies and models have been applied to both static and dynamic gesture recognition scenarios^32^. However, gesture recognition techniques based on various sensors have an unavoidable drawback of high cost, whereas the cost of vision-based gesture recognition is relatively low, and even vision-based gesture recognition tasks can be carried out through mobile handsets. Many existing models for static gesture recognition, trained on static gesture datasets^33^, tend to perform well only within the confines of those datasets and struggle to achieve satisfactory results in real-world applications. This is primarily due to significant differences between the background noise in the datasets and that encountered in actual application scenarios. To address the impact of such background noise disparities on recognition performance, researchers have increasingly focused on this area and made significant progress in developing effective solutions.

To improve the reliability of gesture recognition in uncontrollable environments and diverse lighting conditions, several studies on feature fusion for gesture recognition have emerged^34^. Google's hand landmark model can extract 21 three-dimensional key points of the hand from a single RGB hand image. Similar to gesture images, the data of these 21 key points can be used as features for training gesture recognition models^35^. Refat Khan Pathan et al.^36^ obtained 96.29% accuracy for the image and 98.42% accuracy for the hand features by testing the hand image and the 21 key points features of the hand separately from the "ASL Finger Spelling"^33^ dataset. They then fused the two into a multi-head convolutional network and obtained a test accuracy of 98.98 percent, which was better than the two data alone. This research confirms that incorporating additional feature data, such as the hand key points unaffected by background noise, can effectively enhance the testing accuracy of the model. However, this approach requires prior extraction of the hand key points using Google's hand landmark model before feeding them into the model for further prediction. This certainly adds to the work of preprocessing the image.

For common RGB gesture images, color-based features provide the hand surface texture, but they may not be robust to uneven lighting and complex backgrounds, resulting in the deterioration of vision-based gesture recognition^11^. To reduce the effect of lighting and complex background on model training, SHIH-HUNG YANG et al.^37^ used RGB images and depth images^38^ for model training and proposed a two-path depth-aware attention network model to extract discriminative features while suppressing the effect of color and depth mismatch, and the final model was tested on the "ASL The final model obtained a test accuracy of 93.53% on the "ASL Finger Spelling" dataset. However, this method requires the use of a depth camera to acquire depth images, which is not possible with the mainstream cameras currently in use, and this is not conducive to the direct use of this method by the general public. Dou et al.^39^ introduce a novel approach for foreground detection that utilizes CNNs to address the challenges associated with background subtraction. Experimental results demonstrate the effectiveness of their proposed method. Braham et al.^31^ propose an algorithm for background subtraction that leverages spatial features learned using CNNs. Their model adopts a single background image for background modeling and employs a scene-specific training dataset to train CNNs. These CNNs effectively learn to subtract the background from input image patches. However, these methods either have low gesture recognition performance or their models are too complex to be deployed on portable devices, hindering the application of gesture recognition.

To address these issues, inspired by Braham et al.^31^, we proposed a dual-path background erasing deep convolutional neural network (DPCNN). The input image without much preprocessing operation, and directly put the input data into the DPCNN for training, unlike the exiting methods^37,40^ that do the work of extracting hand features, our two paths do different work. The first path extracts the overall features and the second path extracts the background features (eliminating the hand features) and uses the overall features extracted by the first path to gradually eliminate the background features extracted by the second path to achieve the work of obtaining the hand features. The main goal is not to use the previous CNN strategy of extracting hand features for image recognition, but to extract hand features by eliminating the simpler hand features relative to the background and subtracting the results of the convolution of the two paths. This approach has been experimentally validated to have better recognition capabilities in the test set.

## Materials and methods

### Dataset description

**Figure 1:**
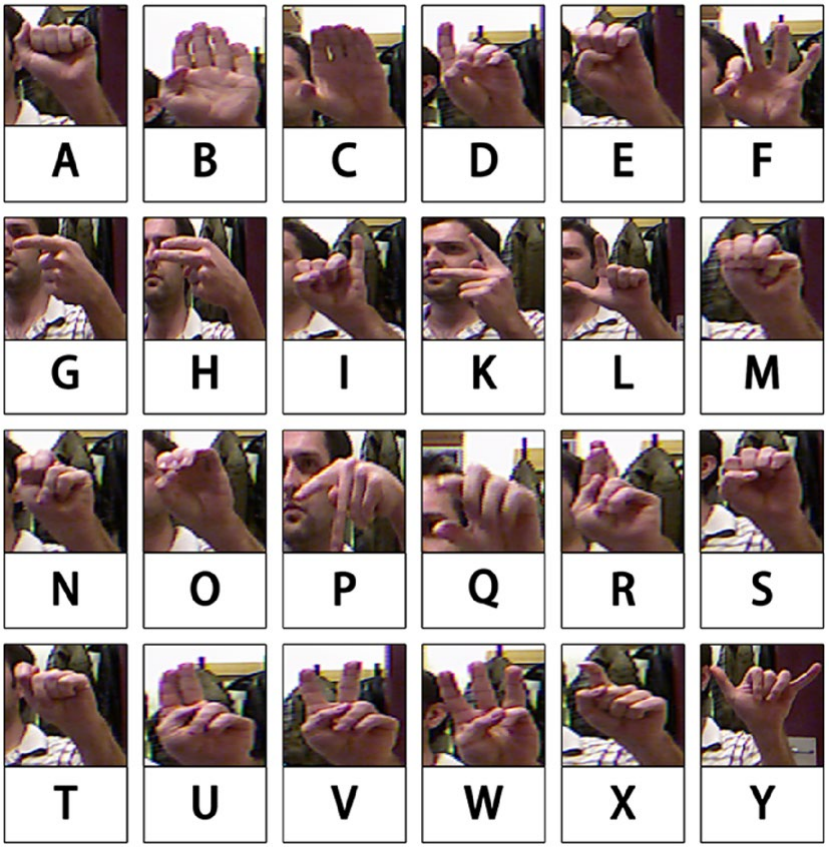
Sample data from 24 categories from filming crew A.

Typical gesture datasets have similar or uniform backgrounds, which allows the model to perform well on the dataset, however, it may not work well in real-world applications. To demonstrate the superior generalization ability of our model, we chose the "ASL Finger Spelling" dataset^41^, which has a more complex background. The dataset consists of 24 alphabetic categories (J and Z are excluded because they are dynamic gestures), captured by five different people, with each participant capturing more than 500 RGB images for each category, and the corresponding depth images are provided in the dataset, where each gesture is photographed from a different angle. We will only use the RGB images from these in our study and not the depth images. The details of the dataset are shown in Table 1 and part of the dataset is displayed in Fig. 1.

**Table 1:** Total number of images taken by each person in the dataset and total number of images.

Participants	Total number of individual participant RGB images	Total number of RGB images
A	12547	65702
B	13826	
C	13393	
D	13154	
E	12782

### Data preprocessing

One of the aims of this study is not to require too much the preprocessing of the image, so we only normalized the original image and resized it to 50 × 50. Data normalization improves the convergence speed and stability of the model.

### Methods

**Figure 2:**
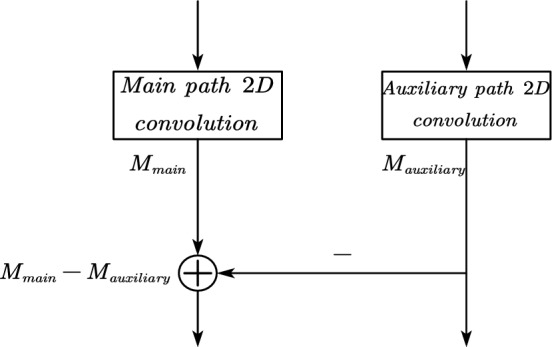
Overall features extracted by primary path minus background features extracted by secondary path to extract hand features.

In this study, we propose a two-path background erasure network model, in which the main path extracts the overall features and the auxiliary path eliminates the hand features to extract the background features and the background features are gradually subtracted from the features extracted by the main path utilizing feature subtraction of the two paths, to effectively eliminate the background information in the image. The structure of the model to eliminate the background is shown in Fig. 2.

The expression for the overall features extracted by the primary path minus the background extracted by the secondary path is:

1 $$ {\text{M}}_{main} = M_{main} - M_{{{\text{auxiliary}}}} $$

$M_{main}$ represents the feature map output by the 2D convolutional layer on the primary path. This map contains gesture features along with background noise that needs to be erased. $M_{{{\text{auxiliary}}}}$ represents the feature map output by the 2D convolutional layer on the secondary path, which contains only background noise. In the Eq. (1), the primary path feature map, with background noise erased, will continue to represent gesture features. Meanwhile, the feature map on the secondary path remains unchanged.

The model can be implemented indirectly through addition, i.e., feeding $M_{main}$ and $- M_{{{\text{auxiliary}}}}$ into the addition layer. By this way, the network model can still be trained end-to-end by the backpropagation algorithm. To evaluate our approach, we conducted comparative experiments with a single-path network model as well as a two-path model with ${\text{M}}_{main} = M_{main} + M_{{{\text{auxiliary}}}}$ connectivity in its structure, and the results show that our model obtains a better performance in the test set with fewer or equal number of parameters, which further validates the better generalization ability of our model structure. The implementation of extracting hand features is achieved by subtracting the background features extracted by the secondary path from the overall features extracted by the primary path as shown in Fig. 2.

### Working procedure

**Figure 3:**
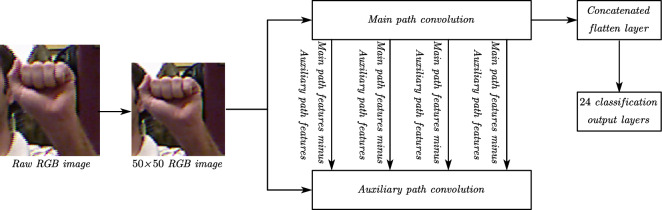
Flow diagram of working procedure.

The model workflow diagram is shown in Fig. 3. The workflow can be divided into two main phases, dataset preprocessing and model construction and training. In the first phase, the dataset is preprocessed, and to facilitate the testing of the model. We save the preprocessed dataset using the Python pickle library, so that it can be quickly read directly from the pickle file when needed. In the second stage, we constructed a lightweight dual-path background erasure network model. The path responsible for extracting the overall features is called the primary path, while the path responsible for extracting the background features is called the secondary path. The primary path will gradually use the features extracted in the secondary path to erase the background, and ultimately to achieve the purpose of extracting hand features.

**Figure 4:**
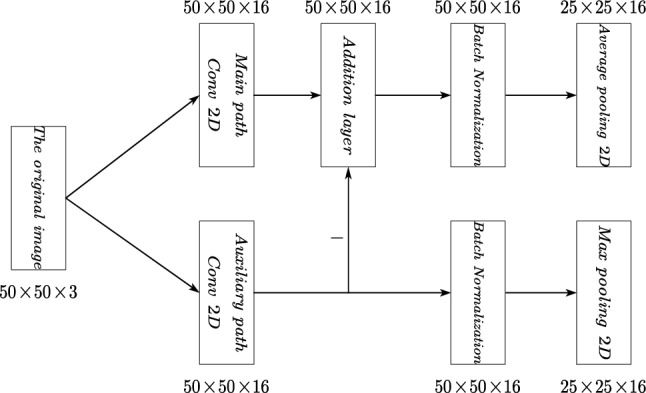
The model's first structure part.

The model's first structure part is shown in Fig. 4, and the rest of the model is similar. The main path consists of a 2D Convolution layer, an Addition layer, a Batch Normalization layer (BN layer), and an Average Pooling layer. The size of input is 50*50*3, and a 50*50*16 feature map is obtained that carries the overall features of the image. These feature maps are then input into the Addition layer and subtracted from the 50*50*16 feature map of the auxiliary path. As a result, the main path obtains a 50*50*16 feature map that has been partially removed from the background noise. To normalize the data, a Batch Normalization (BN) layer is used. Then Average Pooling is chosen for the main path because it is suitable for tasks that focus on the overall features of the data, which is consistent with extracting the overall features. Max Pooling is chosen for the auxiliary path because it is suitable for extracting the most salient features of a local region that helps to extract the background features.

### Model building

The primary path contains 2D convolutional layers with output channel numbers of 16, 32, 32, 16, ReLU layer, BatchNormal layer, and mean pooling layer. The network structure of the auxiliary path is the same as the main path, except for using a max pooling.

**Figure 5:**
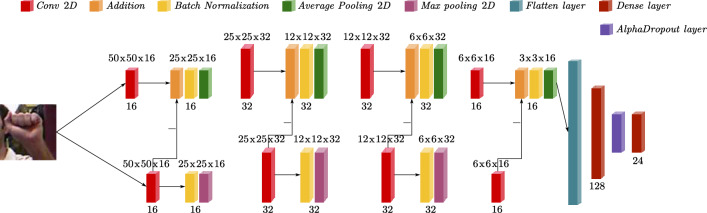
Architecture of the DPCNN. The superscript indicates the shape of the output and the subscript indicates the number of filters.

To guide the auxiliary path to erase the hand features and extract the background features, we subtract the result of each 2D convolutional layer in the primary path from the result of the corresponding 2D convolutional layer in the auxiliary path. And the primary pathway subtracts the background features four times. Next, the output of the primary path is flattened into a one-dimensional tensor and then passes through a fully connected layer with an output unit of 128, and an AlphaDropout layer. Finally, the fully connected layer with 24 output units is used as the output layer and the Softmax activation function is used. The architecture of the model is shown in Fig. 5 and the details of the model are shown in Table 2.

**Table 2:** DPCNN model architecture.

Layer (type)	Output shape	Param #	Connected to
input_1 (InputLayer)	[(None, 50, 50, 3)]	0	
input2_c1 (Conv2D)	(None, 50, 50, 16)	448	input_1[0][0]
input1_c1 (Conv2D)	(None, 50, 50, 16)	448	input_1[0][0]
tf.math.negative (TFOpLambda)	(None, 50, 50, 16)	0	input2_c1[0][0]
add (Add)	(None, 50, 50, 16)	0	input1_c1[0][0]
			tf.math.negative[0][0]
batch_normalization_1 (BatchNormalization)	(None, 50, 50, 16)	64	input2_c1[0][0]
batch_normalization(BatchNormalization)	(None, 50, 50, 16)	64	add[0][0]
max_pooling2d (MaxPooling2D)	(None, 25, 25, 16)	0	batch_normalization_1[0][0]
average_pooling2d (AveragePooling2D)	(None, 25, 25, 16)	0	batch_normalization[0][0]
input2_c2 (Conv2D)	(None, 25, 25, 32)	4640	max_pooling2d[0][0]
input1_c2 (Conv2D)	(None, 25, 25, 32)	4640	average_pooling2d[0][0]
tf.math.negative_1 (TFOpLambda)	(None, 25, 25, 32)	0	input2_c2[0][0]
add_1 (Add)	(None, 25, 25, 32)	0	input1_c2[0][0]
			tf.math.negative_1[0][0]
batch_normalization_3(BatchNormalization)	(None, 25, 25, 32)	128	input2_c2[0][0]
batch_normalization_2(BatchNormalization)	(None, 25, 25, 32)	128	add_1[0][0]
max_pooling2d_1 (MaxPooling2D)	(None, 12, 12, 32)	0	batch_normalization_3[0][0]
average_pooling2d_1 (AveragePooling2D)	(None, 12, 12, 32)	0	batch_normalization_2[0][0]
input2_c3 (Conv2D)	(None, 12, 12, 32)	9248	max_pooling2d_1[0][0]
input1_c3 (Conv2D)	(None, 12, 12, 32)	9248	average_pooling2d_1[0][0]
tf.math.negative_2 (TFOpLambda)	(None, 12, 12, 32)	0	input2_c3[0][0]
add_2 (Add)	(None, 12, 12, 32)	0	input1_c3[0][0]
			tf.math.negative_2[0][0]
batch_normalization_5(BatchNormalization)	(None, 12, 12, 32)	128	input2_c3[0][0]
batch_normalization_4 (BatchNormalization)	(None, 12, 12, 32)	128	add_2[0][0]
max_pooling2d_2 (MaxPooling2D)	(None, 6, 6, 32)	0	batch_normalization_5[0][0]
average_pooling2d_2 (AveragePooling2D)	(None, 6, 6, 32)	0	batch_normalization_4[0][0]
input2_c4 (Conv2D)	(None, 6, 6, 16)	4624	max_pooling2d_2[0][0]
input1_c4 (Conv2D)	(None, 6, 6, 16)	4624	average_pooling2d_2[0][0]
tf.math.negative_3 (TFOpLambda)	(None, 6, 6, 16)	0	input2_c4[0][0]
add_3 (Add)	(None, 6, 6, 16)	0	input1_c4[0][0]
			tf.math.negative_3[0][0]
batch_normalization_6(BatchNormalization)	(None, 6, 6, 16)	64	add_3[0][0]
average_pooling2d_3 (AveragePooling2D)	(None, 3, 3, 16)	0	batch_normalization_6[0][0]
flatten (Flatten)	(None, 144)	0	average_pooling2d_3[0][0]
dense (Dense)	(None, 128)	18560	flatten[0][0]
alpha_dropout (AlphaDropout)	(None, 128)	0	dense[0][0]
dense_1 (Dense)	(None, 24)	3096	alpha_dropout[0][0]
Total params: 60,280			
Trainable params: 59,928			
Non-trainable params: 352			

### Training and testing

**Figure 6:**
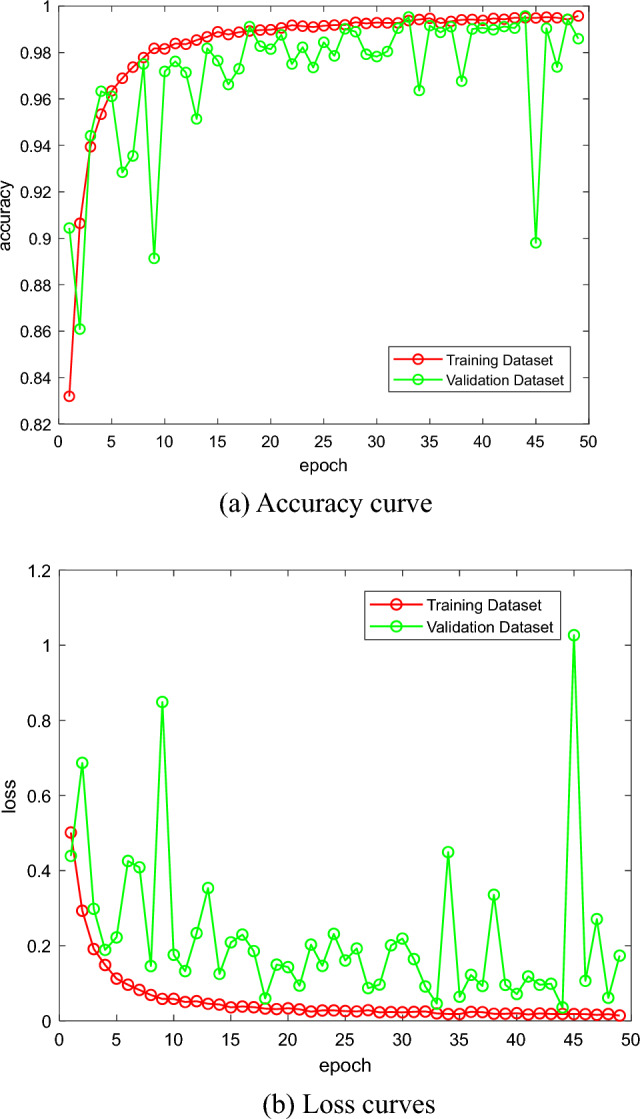
Curves of change in accuracy and loss over 50 rounds.

Before training, the dataset was partitioned into subsets: 39,409 images for the training set, 13,142 images for the validation set, and 13,151 images for the testing set. The partitioning of the images within each category for each image capture participant was done using the same ratio, ensuring a reasonable distribution of the images. In model training, we use the cross-entropy loss function to compute the loss and the Adam optimizer with the learning rate set to 0.001, five different random seeds are used for training, and the average of the test results is taken as the final result. The model was trained using an early stopping strategy with 50 rounds of training. The loss change curves as well as the accuracy change curves of the model on the training and validation sets during the training process are shown in Fig. 6, and the performance of the model on the test set under different random seeds is shown in Table 3.

**Table 3:** Loss and accuracy of models trained under five different random seeds on the test set.

	7	8	9	10	11	Averages
Lose	0.058	0.066	0.047	0.056	0.031	0.052
Accuracy	99.53%	98.44%	99.48%	99.51%	99.65%	99.52%

The first row in the table represents 5 different random seeds.

**Figure 7:**
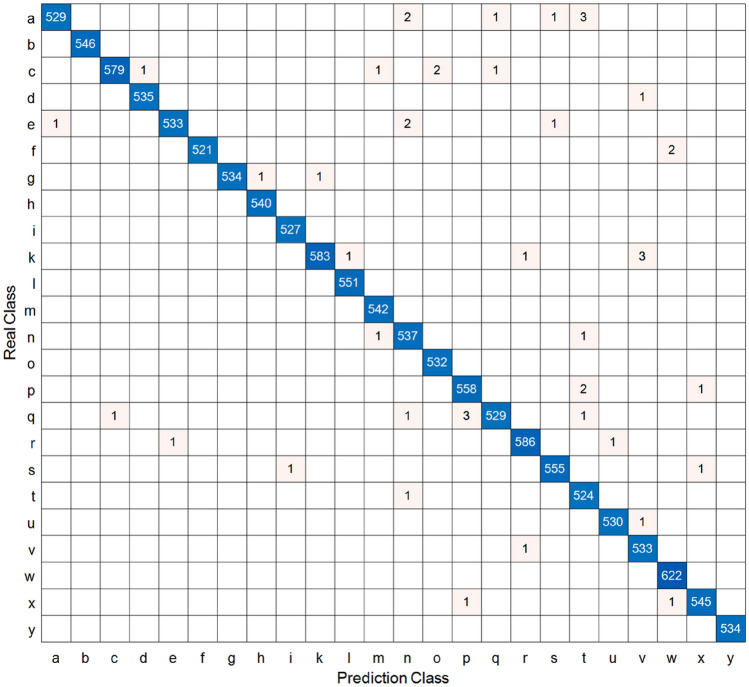
Confusion matrix obtained from the model's prediction results on the test set, with blanks denoting zeros.

To be able to perform a comprehensive evaluation of the trained model, we computed the precision, recall, and F1-score of the model on the test set. To display the prediction results on the test set more intuitively and to facilitate the computation of these evaluation metrics, we also generated the confusion matrix. The confusion matrix contains four values: TP, TN, FP, and FN. TP means predicting positive samples as positive samples, also known as true; TN means predicting negative samples as negative samples, also known as true-negative; FP means predicting negative samples as positive samples, also known as false-positive; and FN means predicting positive samples as negative samples, also known as false-negative. The confusion matrix is shown in Fig. 7 and the three assessment metrics are shown in Table 4, and the mathematical formulas for calculating the three assessment metrics are shown below:

2 $$ {\text{P}}ercision = \frac{TP}{{TP + FP}} $$

3 $$ {\text{Re}} call = \frac{TP}{{TP + FN}} $$

**Table 4:** Precision, Recall, and F1-Score on the test set.

Categories	Precision	Recall	F1-Score	Number of pictures
A	0.998	0.987	0.992	536
B	1.000	1.000	1.000	546
C	0.998	0.991	0.995	584
D	0.998	0.998	0.998	536
E	0.998	0.993	0.995	537
F	1.000	0.996	0.998	523
G	1.000	0.996	0.998	536
H	0.998	1.000	0.999	540
I	0.998	1.000	0.999	ta527
K	0.998	0.991	0.995	588
L	0.998	1.000	0.999	551
M	0.996	1.000	0.998	542
N	0.989	0.996	0.993	539
O	0.996	1.000	0.998	532
P	0.993	0.995	0.994	561
Q	0.996	0.989	0.992	535
R	0.997	0.997	0.997	588
S	0.996	0.996	0.996	557
T	0.987	0.998	0.992	525
U	0.998	0.998	0.998	531
V	0.991	0.998	0.994	534
W	0.995	1.000	0.998	622
X	0.996	0.996	0.996	547
Y	1.000	1.000	1.000	534
Micro average	0.997	0.997	0.997	13151
Macro average	0.997	0.997	0.997	13151
Weighted average	0.997	0.997	0.997	13151

The F1 value (F1-score) represents the reconciled mean of precision and recall and is calculated as:

4 $$ F1 = \frac{2}{{\frac{1}{P} + \frac{1}{R}}} = \frac{2 \times P \times R}{{P + R}} $$

### Experiments

For the task of gesture recognition, many approaches currently exist^25,26,48,49^. However, existing methods usually require complex and time-consuming preprocessing operations or the use of a depth camera to acquire depth images to extract features that are not affected by background interference or are less affected by background noise. Instead of adopting the traditional method of extracting hand features using a CNN network, we propose a new method that uses background erasure to reduce the effect of background noise by employing a convolutional network with dual paths. Table 5 shows the recognition performance of different methods. From Table 5, we can see that the proposed model achieved the highest recognition accuracy. We attribute the excellent performance of the DPCNN to the ability of effectively remove noise. To validate our hypothesis, we conducted experiments using branch addition instead of subtraction.

**Table 5:** Selected past research results on static gesture recognition.

Specificities	Dataset	Accuracy (%)
skeletonization algorithm + CNN^42^	ASK gesture database	96.01
Dual-path depth-aware attention network^37^	ASL Finger spelling dataset	93.53
Color moment + Hu moment + Gray Level Cooccurrence Matrix + SVM^43^	American sign language	87.00
media-pipe + SVM + GBM^44^	ASL Finger spelling dataset	98.45
CNN^45^	HUST-ASL Dataset	98.93
multi‑headed CNN^36^	ASL Finger spelling dataset	98.98
CNN^46^	IPN Hand dataset	87.5
Attention based graph^47^	SHREC’17	97.01
DPCNN	ASL Finger spelling dataset	99.52

To further validate the effectiveness of background elimination in gesture recognition, we conducted a comparative experiment by replacing the key "subtraction" operation in the model with an "addition" operation, i.e., ${\text{M}}_{main} = M_{main} - M_{{{\text{auxiliary}}}}$ with ${\text{M}}_{main} = M_{main} + M_{{{\text{auxiliary}}}}$. This approach provides a more intuitive and effective demonstration of the effectiveness of our model. Except for the model structure, we maintained consistent experimental conditions, including the same dataset, five random seeds^7–11^, learning rate, and other experimental settings. The final average test accuracy obtained is shown in Table 6. From Table 6, we can see that the branch subtraction of the model is superior to the addition.

**Table 6:** Performance of the trained model on the test set with identical external conditions except for the different expressions in the connecting part of the primary and secondary paths.

Modelling	Dataset	Average loss	Average accuracy (%)
${\text{M}}_{main} = M_{main} + M_{{{\text{auxiliary}}}}$	ASL	0.073	99.31
${\text{M}}_{main} = M_{main} - M_{{{\text{auxiliary}}}}$	ASL	0.052	99.52

ASL stands for American Sign Language.

The learning curves in Fig. 6 show that the validation accuracy of the best results from multiple random seeds consistently approaches or even exceeds the training accuracy. This reflects the high generalization ability of our model as it successfully mitigates the effect of background noise on recognition accuracy. The results in Table 4 show that our model performs well on various evaluation metrics. Relative to the methods of Refat Khan Pathan et al.^36^ and SHIH-HUNG YANG et al.^37^, our model yields a better average test accuracy of 99.52%, which indicates that our proposed model is effective. Compared with these methods, the proposed model is lightweight.

**Figure 8:**
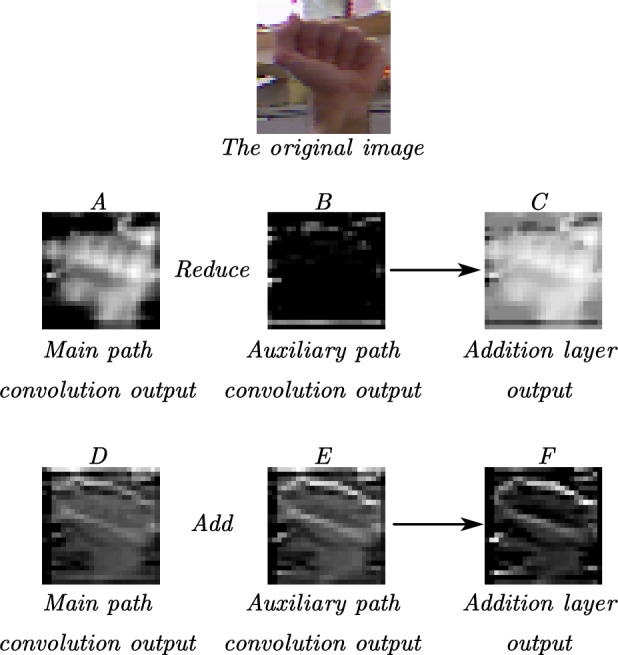
representative feature maps with better-preserved hand features in the feature maps output from the second addition layer of the model.

To further analyze the reasons for the excellent performance of the proposed model, some feature maps were visualized (as shown in Fig. 8). We input a test set image to the model, and to obtain a hand feature map that is not too abstract and easily recognizable by the human eye. We obtained representative feature maps with better-preserved hand features in the feature maps output from the second addition layer of the model, as shown in Fig. 8. The feature maps in the middle three sheets (A, B, C) are displayed. The output image A from the convolution in the main path before the second addition layer. The output image B from the convolution in the auxiliary path before the second addition layer, and the output image C before the second addition layer. The output images that correspond to the convolution output image D in the main path. The convolution output image E in the auxiliary path, and the convolution output image E in the main path, as well as the convolution output image D in the auxiliary path. They are all in front of the second Addition layer. Additionally, the output images that correspond to the convolution output image E in the auxiliary path and the output image F of the second Addition layer are also in front of the second Addition layer.

Based on Image A and the original image, it is evident that the hand image features have been extracted. Similarly, Image B and the original image show that Image B has extracted the background features. Image C was obtained by subtracting image B from image A, preserving the hand features and further weakening the background features. Based on images D, E, and F, the three feature maps appear very similar and still contain the prominent background features. The images provide two pieces of information. Firstly, the model using subtraction can extract background features on the auxiliary path to some extent and then eliminate them by subtracting the convolutional output of the auxiliary path from the convolutional output of the main path. Secondly, the model using subtraction outperforms the model using addition. However, in the case of noisy images, the hand image may become more complex due to a significant amount of noise in comparison to the background image. In this case, the auxiliary path for eliminating simpler hand features has lost its original purpose, which may cause the model's recognition ability a decrease.

From Table 5, it can be seen that although the DPCNN have achieved the best performance, the advantage is not very obvious. However, the purpose of comparison is not to prove that our method is the best, but to demonstrate that our method is effective and feasible. More importantly, compared with using various professional gesture recognition devices (such as Kinect sensor^12^, smart gloves^8^, etc.), the proposed method in this study is very simple. The parameter sizes of the models proposed by Pathan et al.^36^ and Yang et al.^37^ are 1.88 M and 21.24 M, respectively. However, the parameter sizes of the DPCNN is 0.06 M. Therefore, the proposed model can be used in small terminals with limited resources.

## Conclusion

Our dual-path background erasure network model extracts discriminative features in RGB images for gesture recognition and avoids the influence of different background noises on the model recognition by simple preprocessing of RGB images, which leads to a better generalization ability of the model in practical applications. Our model improves the previous feature extraction method, the previous method directly extracts the hand features, this method can be understood as erasing the background features, but for the complex background features, this method makes it difficult to maintain good performance in practical applications. Therefore, we break through this idea and propose to erase the simpler hand features relative to the background features, which significantly improves the generalization ability of the model. The dual-path background erasure network model employs the design idea of primary and secondary paths to achieve our goal. The primary path is used to extract the overall features, while the secondary path is used to erase the hand features and extract the background features. By subtracting the features extracted by the primary path from the features extracted by the secondary path, we gradually eliminate the background features and highlight the extraction of hand features. The two-path background erasure network model outperforms current state-of-the-art methods on the ASL Finger Spelling dataset. In future work, we will expand the scope of our research, test more different types of datasets, and try new model concepts to further improve the ability of background feature extraction by auxiliary paths.

## Data Availability

The dataset used in this article is Kaggle's publicly available dataset (ASL Fingerspelling Images (RGB & Depth)) addressed as: https://www.kaggle.com/datasets/mrgeislinger/asl-rgb-depth-fingerspelling-spelling-it-out.

## References

[1] Xiu-qin A (2006). The art of language communication between nurses and patients. China Med. Herald.

[2] Hardini S, Sitohang R (2019). The use of language as a socialcultural communication. J. Littera Fakultas Sastra Darma Agung.

[3] Anderson R, Wiryana F, Ariesta MC (2017). Sign language recognition application systems for deaf-mute people: a review based on input-process-output. Proc. Comp. Sci..

[4] Rastgoo, R., Kiani, K., Escalera, S., et al. Sign language production: A review. *Proceedings of the IEEE/CVF conference on computer vision and pattern recognition*. 3451–3461 (2021).

[5] Sahoo AK, Mishra GS, Ravulakollu KK (2014). Sign language recognition: State of the art. ARPN J. Eng. Appl. Sci..

[6] Rautaray SS, Agrawal A (2015). Vision based hand gesture recognition for human computer interaction: A survey. Artif. Intell. Rev..

[7] Koller, O. Quantitative survey of the state of the art in sign language recognition. arXiv preprint arXiv:2008.09918, (2020).

[8] Wen F, Zhang Z, He T (2021). AI enabled sign language recognition and VR space bidirectional communication using triboelectric smart glove. Nat. Commun..

[9] Ahmed MA, Zaidan BB, Zaidan AA (2018). A review on systems-based sensory gloves for sign language recognition state of the art between 2007 and 2017. Sensors.

[10] Estrela, B., Cámara-Chávez, G., Campos, M. F, et al. Sign language recognition using partial least squares and RGB-D information. *Proceedings of the IX Workshop de Visao Computacional*, WVC. (2013).

[11] Wang Yong Wu, Jin-jun T-S (2019). Multi-dimensional parameter gesture recognition algorithm based on FMCW radar. J. Elect. Inf. Techn..

[12] Raghuveera T, Deepthi R, Mangalashri R (2020). A depth-based Indian sign language recognition using microsoft kinect. Sādhanā.

[13] Tompson J, Stein M, LeCun Y (2014). Real-time continuous pose recovery of human hands using convolutional networks. ACM Trans. Graph..

[14] Cao J, Yu S, Liu H (2016). Hand posture recognition based on heterogeneous features fusion of multiple kernels learning. Multimed. Tools Appl..

[15] Sadeddine K, Chelali FZ, Djeradi R (2021). Recognition of user-dependent and independent static hand gestures: Application to sign language. J. Visual Commun. Image Repres..

[16] Iandola, F. N., Han, S. & Moskewicz, M. W. et al. SqueezeNet: AlexNet-level accuracy with 50x fewer parameters and< 0.5 MB model size. arXiv preprint arXiv:1602.07360, (2016).

[17] Howard, A. G., Zhu, M. & Chen, B. et al. Mobilenets: Efficient convolutional neural networks for mobile vision applications. arXiv preprint arXiv:1704.04861, (2017).

[18] Sandler, M., Howard, A. & Zhu, M. et al. Mobilenetv2: Inverted residuals and linear bottlenecks. *Proceedings of the IEEE conference on computer vision and pattern recognition*. pp 4510–4520, (2018).

[19] Zimmermann, C., Brox, T. Learning to Estimate 3D Hand Pose from Single RGB Images. *IEEE International Conference on Computer Vision, Venice, Italy*, pp 4913–4921, (2017).

[20] Chen, Y., Ma, H. & Kong, D. et al. Nonparametric Structure Regularization Machine for 2D Hand Pose Estimation. *IEEE Winter Conference on Applications of Computer Vision, Colorado, USA*, pp 370–379, (2020).

[21] Kwolek B, Baczynski W, Sako S (2021). Recognition of JSL fingerspelling using deep convolutional neural networks. Neurocomputing.

[22] Huang, J., Zhou, W. & Li H. et al. Atten⁃tion based 3D-CNNs for large-vocabulary sign language recognition. *IEEE Transactions on Circuits and Sys⁃tems for Video Technology*, 29(9), pp 2822–2832, (2019).

[23] Koller, O., Camgoz, C. & Ney H, et al. Weakly supervised learning with multi-stream CNN-LSTM-HMMs to discov⁃er sequential parallelism in sign language videos. *IEEE Transactions on Pattern Analysis and Machine Intelligence*, 42(9), pp 2306–2320, (2019). 10.1109/TPAMI.2019.2911077 30990421

[24] Liao, Y., Xiong, P. & Min, W. et al. Dynamic sign language recognition based on video sequence with BLSTM-3D re⁃sidual networks. IEEE Access, pp 38044–38054, (2019).

[25] Huang S, Mao C, Tao J (2018). A novel chinese sign lan⁃guage recognition method based on keyframe-centered clips. IEEE Signal Proc, Lett..

[26] Pu, J., Zhou, W. & Li, H. Sign language recognition with multi-modal features. *Pacific Rim Conference on Multimedia. Springer International Publishing*, pp 252–261, (2016).

[27] Liu J, Wang X, Wang C (2023). Temporal decoupling graph convolutional network for skeleton-based gesture recognition. IEEE Trans. Multimed..

[28] Jin B, Ma X, Zhang Z (2023). Interference-robust millimeter-wave radar-based dynamic hand gesture recognition using 2D CNN-transformer networks. IEEE Internet Things J..

[29] Xie P, Cui Z, Du Y (2023). Multi-scale local-temporal simi-larity fusion for continuous sign language recogni- tion. Pattern Recog..

[30] Yang K, Kim M, Jung Y (2024). Hand gesture recognition using FSK radar sensors. Sensors.

[31] Braham, M., Van Droogenbroeck, M. Deep background subtraction with scene-specific convolutional neural networks. *2016 international conference on systems, signals and image processing (IWSSIP)*. IEEE, pp 1–4 (2016).

[32] Ye, Y., Tian, Y. & Huenerfauth, M. et al. Recognizing american sign language gestures from within continuous videos. *Proceedings of the IEEE Conference on Computer Vision and Pattern Recognition Workshops*. Pp 2064–2073, (2018).

[33] Pugeault, N., Bowden, R. Spelling it out: Real-time ASL fingerspelling recognition. *2011 IEEE International conference on computer vision workshops (ICCV workshops)*. IEEE, pp 1114–1119, (2011).

[34] Rahim MA, Islam MR, Shin J (2019). Non-touch sign word recognition based on dynamic hand gesture using hybrid segmentation and CNN feature fusion. Appl. Sci..

[35] Sen-bao W, Jin-Xiao Y, Zi-ang W (2023). Research on gesture Recognition based on hand keypoint detection. Comp. Telecommun..

[36] Pathan RK, Biswas M, Yasmin S (2023). Sign language recognition using the fusion of image and hand landmarks through multi-headed convolutional neural network. Sci. Rep..

[37] Yang SH, Chen WR, Huang WJ (2020). Ddanet: Dual-path depth-aware attention network for fingerspelling recognition using rgb-d images. IEEE Access.

[38] Chu-qing C, Rui-feng Li, Li-jun Z (2012). Gesture recognition method based on depth image technology. Comp. Eng..

[39] Dou J, Qin Q, Tu Z (2019). Background subtraction based on deep convolutional neural networks features. Multimed. Tools Appl..

[40] Gao, Q., Sun, L. & Han, C. et al. American Sign Language fingerspelling Recognition Using RGB-D and DFANet. *2022 China Automation Congress (CAC)*. IEEE, pp 3151-3156, (2022).

[41] Pugeault, N., Bowden, R. Spelling It Out: Real-Time ASL Fingerspelling Recognition. In *Proceedings of the 1st IEEE Workshop on Consumer Depth Cameras for Computer Vision, jointly with ICCV*'. 10.1109/ICCVW.2011.6130290. (2011).

[42] Jiang D, Li G, Sun Y (2019). Gesture recognition based on skeletonization algorithm and CNN with ASL database. Multimed. Tools Appl..

[43] Kaslay, S., Kesarkar, T. & Shinde K. ASL Gesture Recognition Using Various Feature Extraction Techniques and SVM. *Int. Res. J. Eng. Techn.*, (2020).

[44] Shin J, Matsuoka A, Hasan MAM (2021). American sign language alphabet recognition by extracting feature from hand pose estimation. Sensors.

[45] Sahoo JP, Prakash AJ, Pławiak P (2022). Real-time hand gesture recognition using fine-tuned convolutional neural network. Sensors.

[46] Peral M, Sanfeliu A, Garrell A (2022). Efficient hand gesture recognition for human-robot interaction. IEEE Robot Autom. Lett..

[47] Miah ASM, Hasan MAM, Shin J (2023). Dynamic hand gesture recognition using multi-branch attention based graph and general deep learning model. IEEE Access.

[48] Adithya V, Rajesh R (2020). A deep convolutional neural network approach for static hand gesture recognition. Proc. Comp. Sci..

[49] Ren, Z., Yuan, J. & Zhang, Z. Robust hand gesture recognition based on finger-earth mover's distance with a commodity depth camera. *Proceedings of the 19th ACM international conference on Multimedia*. pp 1093–1096, (2011).

